# *Coix lacryma-jobi var. ma-yuen* Stapf sprout extract has anti-metastatic activity in colon cancer cells in vitro

**DOI:** 10.1186/s12906-017-1990-y

**Published:** 2017-11-06

**Authors:** Eun Suk Son, Young Ock Kim, Chun Geon Park, Kyung Hun Park, Sung Hwan Jeong, Jeong-Woong Park, Se-Hee Kim

**Affiliations:** 1 Department of Internal Medicine, Gachon University Gil Medical Center, 21 Namdong-daero 774 beon-gil, Namdong-gu, Incheon, 405-760 Republic of Korea; 2 Department of Herbal Crop Research, National Institute of Horticultural and Herbal Science, RDA, Cheongju, Chungbuk Republic of Korea; 3 Gachon medical research institute, Gachon University Gil Medical Center, 21 Namdong-daero 774 beon-gil, Namdong-gu, Incheon, 405-760 Republic of Korea

**Keywords:** *Coix*, Colon cancer, Metastasis, Invasion, Hypoxia

## Abstract

**Background:**

*Coix lacryma-jobi var. ma-yuen* (Rom.Caill.) Stapf has been used in China as an herbal medicine. Many studies of this plant have reported anti-proliferative and apoptotic activities on human cancer cell lines. Therefore, this study of the anti-metastatic effect of *Coix lacryma-jobi var. ma-yuen* Stapf sprout extract (CLSE) in colorectal cancer cells may provide a scientific basis for exploring anti-cancer effects of edible crops.

**Methods:**

To evaluate the effect of CLSE on cell proliferation and signaling, we performed a Cell Counting Kit-8 (CCK-8) assay in HCT116 cells and used western blot analysis. Furthermore, scratch-wound healing, transwell migration, matrigel invasion, and adhesion assays were conducted to elucidate the anti-metastatic effects of CLSE under hypoxic conditions in colon cancer cells.

**Results:**

First, CLSE decreased deferoxamine (DFO)-induced migration of colon cancer cells by 87%, and blocked colon cancer cell migration by 80% compared with hypoxia control cells. Second, CLSE treatment resulted in a 54% reduction in hypoxia-induced invasiveness of colon cancer cells, and 50% inhibition of adhesive potency through inactivation of the extracellular signal-regulated kinase (ERK) 1/2 and protein kinase b (AKT) pathways. Third, conditioned medium collected from CLSE-treated HCT116 cells suppressed tube formation of human umbilical vein endothelial cells (HUVECs) by 91%.

**Conclusions:**

CLSE inhibited migration, invasion, and adhesion of colon cancer cells and tube formation by HUVECs via repression of the ERK1/2 and AKT pathways under hypoxic conditions. Therefore, CLSE may be used to treat patients with colon cancer.

## Background

Worldwide, colon cancer is one of the most deadly cancers because it is highly metastatic and invasive. An important determinant of the prognosis of cancer patients is the progression of tumor cell metastasis and invasion. The ability for metastasis and invasion enables cancer cells to find new areas of the body to occupy when space and nutrients become limited in their current location. The metastatic cascade can be separated into three processes: invasion, intravasation, and extravasation. First, the process of invasion involves the dissociation of tumor cells from the primary tumor mass and subsequent invasion into the surrounding tissue. Next, intravasation occurs when detached cells are transported via blood vessels to distant sites. Finally, tumor cells interact with endothelial cells to form stronger bonds, and penetrate the endothelium and basement membrane. Consequently, the new tumor cells can proliferate in secondary sites. Therefore, the metastatic spread of tumor tissue requires the growth of a vascular network [1]. The growth of new blood vessels (angiogenesis) is required for primary tumor growth as well as tumor invasion and metastasis [1]. The vasculature that supplies oxygen and nutrients is important for cancer cell survival [2].

Tumor hypoxia results from an imbalance between the oxygen supply and demand due to uncontrolled tumor cell proliferation [3]. Because cancer cells rapidly proliferate, the tumor quickly exhausts the nutrient and oxygen supply from the normal vasculature, and becomes hypoxic. This hypoxic condition upregulates the production of angiogenic factors from hypoxic tumor sites [4]. Therefore, hypoxia signaling can contribute to tumor progression by promoting tumor cell migration, invasion, metastasis, and angiogenesis [5].

*Coix lacryma-jobi var. ma-yuen* (Rom.Caill.) Stapf, which is an important cereal crop for many indigenous groups in upland areas, is characterized by having a similar appearance and taste to rice, with a standing crop comparable with corn. This plant is utilized as a rice alternative, health-promoting staple crop, and as an alternative livelihood and income source through value-added products. An increase in the number of health-conscious individuals has also contributed to the popularity of *Coix,* with the market currently growing due to increased acceptance of this product. *Coix* is largely consumed for household food security as a rice alternative or used to make porridge, champorado, and other recipes.

Previous studies have reported that *Coix* extract has anti-proliferative and apoptotic activities on human lung cancer, histolytic lymphoma, and colon cancer cells, as well as chemopreventive effects on lung cancer in vivo [6–9]. Although a few studies have reported that *Coix* has anti-cancer effects in terms of regulating the proliferation and cell cycle of cancer cells, the effects of *Coix lacryma-jobi var. ma-yuen* Stapf sprout extract (CLSE) on cancer metastasis are unknown. Therefore, this study aimed to explore the anti-cancer effects of CLSE in colorectal cancer cells.

## Methods

### Reagents

CLSE was manufactured in the herbarium of the Herbal Crop Research Institute (Eumseong, Republic of Korea).

Deferoxamine (DFO), Phorbol 12-myristate 13-acetate (PMA), and SC79 were obtained from Sigma-Aldrich (St. Louis, MO, USA). CLSE and DFO were dissolved in water. PMA and SC79 were dissolved in the solvent dimethyl sulfoxide (DMSO).

### CLSE preparation

*Coix* cultivars were obtained from the National Institute of Crop Science (Miryang, Republic of Korea). *Coix* were germinated in a modified commercial soil bed (0.7–1.0 mg/m^3^ soil bulk density, 450–650 mg/L available phosphate, 800–1000 mg/kg nitrogen) (Punong Bed Soil, Gyeongju, Republic of Korea). The germinated *Coix* was grown at 22–23 °C with humidity of 60% in a 900–1000 lx environment. Between 15 and 22 d after germination, young barley leaves about 8–13-cm long were harvested and freeze-dried [10]. We used a water extraction method because most traditional Oriental herbs are decocted in boiling water. In addition, some components are more soluble in water than in organic solvents. Crushed plant materials (200 g each) were extracted three times under reflux with distilled water. The water extracts were combined and lyophilized. The yield was 25% (wt/wt) of the dried *Coix* sprouts. Extracts were stored at −20 °C until usage. A voucher specimen (HPR-208) was deposited in the herbarium of Herbal Crop Research Institute (Eumseong, Republic of Korea).

### Cell lines and cell culture conditions

HCT116 and CCD-18Co cells were obtained from the Korean Cell Line Bank (Seoul, Republic of Korea). Human umbilical vein endothelial cells (HUVECs) were obtained from the Lonza (San Diego, CA, USA). HCT116 cells were cultured in McCoy’s medium (Gibco Cell Culture, Carlsbad, CA, USA) supplemented with 10% fetal bovine serum (FBS) (Gibco) and 1% penicillin-streptomycin (Gibco). CCD-18Co cells were cultured in MEM (Gibco) with 10% FBS (Gibco) and 1% penicillin-streptomycin (Gibco), and were used between passages 5 and 6. HUVECs were grown in EBM-2 (Lonza) supplemented with an EGM™-2 SingleQuots™ kit (Lonza), and used between passages 2 and 4 for experiments. Cells were incubated at 37 °C in a humidified atmosphere with 5% CO_2_. A hypoxia incubator (New Brunswick Scientific, Edison, NJ, USA) containing 1% O_2_, 5% CO_2_, and 94% N_2_ was used to create hypoxic conditions.

### Cell counting Kit-8 (CCK-8) assay

Cells were seeded into 96-well plates and exposed to various concentrations of CLSE for 24–72 h prior to the addition of 10 μL CCK-8 solution (Dojindo Molecular Technologies, Inc., Rockville, MD, USA) containing 2-(2-methoxy-4-nitrophenyl0–3-(4-nitrophenyl)-5-(2,4-disulfophenyl)-2H–tetrazolium, monosodium salt (WST-8) to each well. After 1 h of incubation at 37 °C in a humidified atmosphere with 5% CO_2_, the absorbance was determined at 450 nm.

### Scratch-wound healing assay

Cells were seeded in 60-mm plates and then scratched using a pipette tip after 24-h incubation. After washing with phosphate buffered saline (PBS), cells were incubated in medium containing CLSE and/or 100 μM DFO for 24 h. Images were then obtained at 0 and 24 h with an Olympus CFX41 microscope (Hamburg, Germany) at 40× magnification. The area of cell migration was quantified using ImageJ software (https://imagej.nih.gov/ij/) and the percentage of wound closure was calculated as described previously [11].

### Transwell migration assay

The experiment was performed as described previously [11]. Briefly, cells in CLSE-containing serum-free medium were seeded into the inner chamber with the lower surface coated with 0.2% gelatin. The cells were incubated in normoxic or hypoxic conditions for 24 h. 20% FBS-containing medium in the bottom chamber was used as a chemoattractant. Cells on the upper membrane were wiped off using wet cotton swabs after fixation with methanol and crystal violet staining. Cells on the lower surface were mounted using mounting solution (Vectashield®; Vector Laboratories Burlingame, CA, USA). Stained cells were counted under a light microscope (DP72; Olympus, Hamburg, Germany) and images were taken at 200× magnification. All of the experiments were independently repeated in triplicate.

### Matrigel invasion assay

The matrigel invasion assay was performed as previously described [11]. Briefly, the lower surface were coated with 0.2% gelatin, and then the upper surfaces were coated with Matrigel® (BD Biosciences, San Jose, CA, USA) at 37 °C for 2 h. Cells in CLSE-containing serum-free medium were plated into the inner chamber and incubated in normoxic or hypoxic conditions for 48 h with 20% FBS in the bottom chamber as the chemoattractant. The cells were processed as described for the transwell migration assay. All of the experiments were independently repeated in triplicate.

### Adhesion assay

Cells were treated with or without CLSE and/or DFO for 24 h, suspended in serum-free McCoy’s medium, and then seeded in a 96-well plate that had been pre-coated with Matrigel® (BD Biosciences). After incubation at 37 °C for 90 min, the cells were washed with PBS and treated with 0.5 mg/mL 3-(4,5-Dimethylthiazol-2-yl)-2,5-diphenyltetrazolium bromide (MTT). The absorbance of formazan crystals dissolved in 100 μL DMSO was determined at 570 nm using a microplate reader. The experiments were independently performed in triplicate.

### Western blot analysis

Cells were harvested with lysis buffer [50 mM Tris-HCl (pH 8.0), 0.5% sodium deoxycholate, 150 mM NaCl, 0.1% sodium dodecyl sulfate (SDS), and 1% NP-40]. Antibodies to p65, phospho-p65, extracellular signal-regulated kinase (ERK) 1/2, phospho-ERK1/2, protein kinase B (AKT), phospho-AKT, c-Jun N-terminal kinase (JNK), phospho-JNK, p38, phospho-p38, Signal transducer and activator of transcription 3 (STAT3), and phospho-STAT3 were all purchased from Cell Signaling Technology (Beverly, MA, USA). β-actin antibody was obtained from Santa Cruz Biotechnology (Santa Cruz, CA, USA). Immunoblot bands were quantified as a ratio of phosphorylated protein/total protein using ImageJ software.

### Generation of conditioned medium

HCT116 cells were treated with or without CLSE (0.5, 1 mg/mL) and/or DFO (100 μM) for 24 h in complete medium, and then the supernatant was saved. The supernatant, called conditioned medium, was filtered and stored at −70 °C.

### Tube formation assay

HUVECs were diluted in conditioned medium and seeded in a 48-well plate that had been pre-coated with Matrigel®. After a 12-h incubation period, images of formed tubules were taken at 40× magnification and quantified by determining the number of branching points. The experiments were independently performed in triplicate.

### Statistical analysis

All data were analyzed with GraphPad Prism® software using a two-tailed Student *t* test. *P* values less than 0.05 were significantly considered.

## Results

### CLSE inhibits colon cancer cell migration under hypoxic conditions

**Fig. 1:**
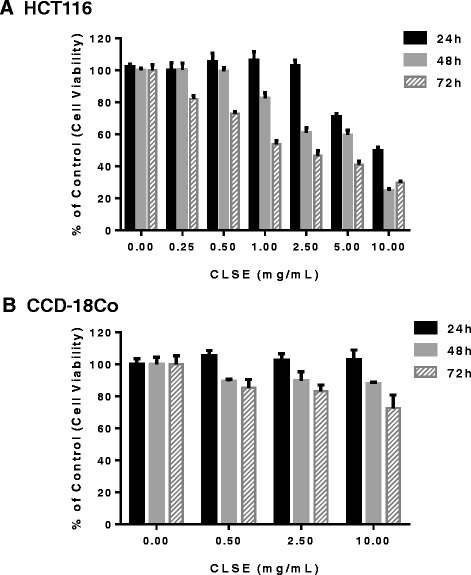
Cytotoxic effect of CLSE on HCT116 and CCD-18Co cells. **a, b**. A Cell Counting Kit (CCK)-8 assay was used to measure HCT116 and CCD-18Co cells proliferation for 24–72 h after treatment with various concentrations of CLSE. Data are mean ± SD. CLSE, *Coix lacryma-jobi var. ma-yuen* Strapt sprout extract; SD, Standard Deviation

**Fig. 2:**
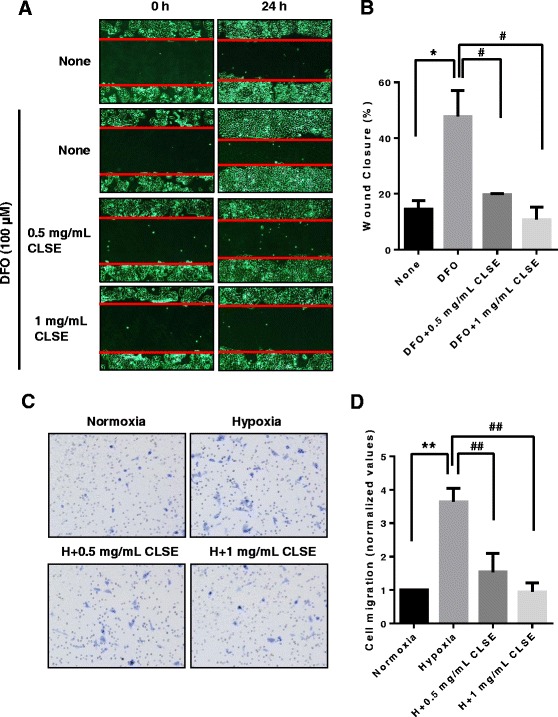
Effect of CLSE on the migratory ability of HCT116 cells under hypoxic conditions. **a**. Representative scratch-wound images showing the effect of CLSE on the healing ability of HCT116 cells (magnification: ×40). After with treatment DFO (100 μM) and CLSE (0.5, 1 mg/mL) for 24 h, the migratory ability was analyzed by scratch-wound healing assay. **b**. Percentage of HCT116 cells that migrated into the wound following CLSE treatment relative to untreated control cells. * *p* < 0.05 versus untreated control group. ^#^ *p* < 0.05 versus DFO-only group. **c**. Representative images showing the effect of CLSE on HCT116 cell migration through a Transwell® chamber membrane (magnification: ×200). After treatment with CLSE (0.5, 1 mg/mL) for 24 h, the migration cells in transwells were stained by crystal violet solution. **d**. Percentage of HCT116 cells that migrated following CLSE treatment relative to control cells (normoxia). ** *p* < 0.01 versus normoxia control group. ^##^ *p* < 0.01 versus hypoxia-only group. Data are mean ± SD (*n* = 3). CLSE, *Coix lacryma-jobi var. ma-yuen* Strapt sprout extract; H, hypoxia; SD, Standard Deviation

To examine effects on the viability of colon cancer cells, HCT116, and of normal cells, CCD-18Co, caused by CLSE treatment, we performed a CCK-8 assay. As shown in Fig. 1, CLSE strongly decreased the viability of HCT116 cells compared to CCD-18Co cells. Next, we performed scratch-wound healing assays to determine if CLSE had an effect on the migratory potency of HCT116 cells. For this assay, we used DFO, a reagent used to simulate hypoxic conditions. As shown in Fig. 2a and b, DFO enhanced the migration of colon cancer cells (*p* < 0.05). However, HCT116 cells co-treated with DFO and CLSE had 47–87% less healing ability than cells treated with DFO only (*p* < 0.05). Furthermore, to confirm the inhibitory effect of CLSE on HCT116 cell migration, we plated CLSE-treated HCT116 cells in a Transwell® chamber and incubated the cells for 24 h under hypoxic conditions. The results showed that CLSE inhibited the migration of hypoxic HCT116 cells in the Transwell® chamber by 48–80% compared with the hypoxia control group (*p* < 0.01) (Fig. 2c, d). These results imply that CLSE has the potential to inhibit HCT116 cell migration under hypoxic conditions.

### CLSE inhibits invasion and adhesion of colon cancer cells in hypoxia

**Fig. 3:**
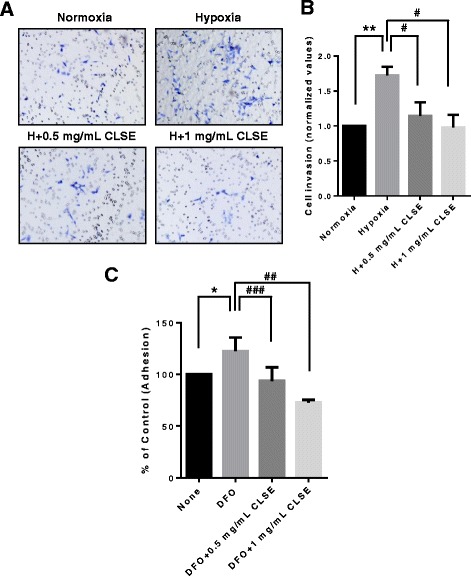
Effect of CLSE on HCT116 cell invasion and migration under hypoxic conditions. **a**. Representative images showing the effect of CLSE on HCT116 cell invasion through a Matrigel®-coated Transwell® chamber membrane (magnification: 200×). After treatment with CLSE (0.5, 1 mg/mL) for 48 h under hypoxic conditions, cells that had invaded the transwells were analyzed through crystal violet staining. **b**. Number of HCT116 cells that invaded following CLSE treatment. The values are normalized to the number of invaded control cells (normoxia). ** *p* < 0.01 versus normoxia control group. ^#^ *p* < 0.05 versus hypoxia-only group. **c**. After treatment with DFO (100 μM) and/or CLSE (0.5, 1 mg/mL) for 24 h, cells were seeded into Matrigel-coated wells and then analyzed using an MTT assay. Number of HCT116 cells that adhered to the Matrigel®-coated plate following CLSE treatment. The values are normalized to the number of adherent control cells (untreated). * *p* < 0.05 versus untreated control group. ^##^ *p* < 0.01, ^###^ *p* < 0.001 versus DFO-only groups. Data are mean ± SD (*n* = 3). CLSE, *Coix lacryma-jobi var. ma-yuen* Stapf sprout extract; H, hypoxia; SD, standard deviation

In cancer cell metastasis, invasion is as important as migration. To investigate the effects of CLSE on colon cancer cell invasion, we performed an invasion assay using a Transwell® coated with Matrigel®. As we showed, hypoxia promoted the invasion of HCT116 cells (*p* < 0.01). However, CLSE-treated cells under hypoxic conditions showed a 21–54% reduction in invasion compared with the hypoxia control group (*p* < 0.05) (Fig. 3a, b).

Metastatic cancer cells have high adhesion potency to migrate to secondary sites for new tumor growth. Therefore, cell adhesion assays are used to examine the metastatic potency of cancer cells. As shown in Fig. 3c, DFO-treated cells had increased adhesion compared with untreated cells (*p* < 0.05); however, cells co-treated with DFO and CLSE showed a 19–50% reduction in adhesive ability compared with cells treated with DFO only (*p* < 0.001, *p* < 0.01). Taken together, these results suggest that CLSE regulates the invasion and adhesion of HCT116 cells under hypoxic conditions.

### CLSE inactivates ERK1/2 and AKT activities in hypoxic conditions

**Fig. 4:**
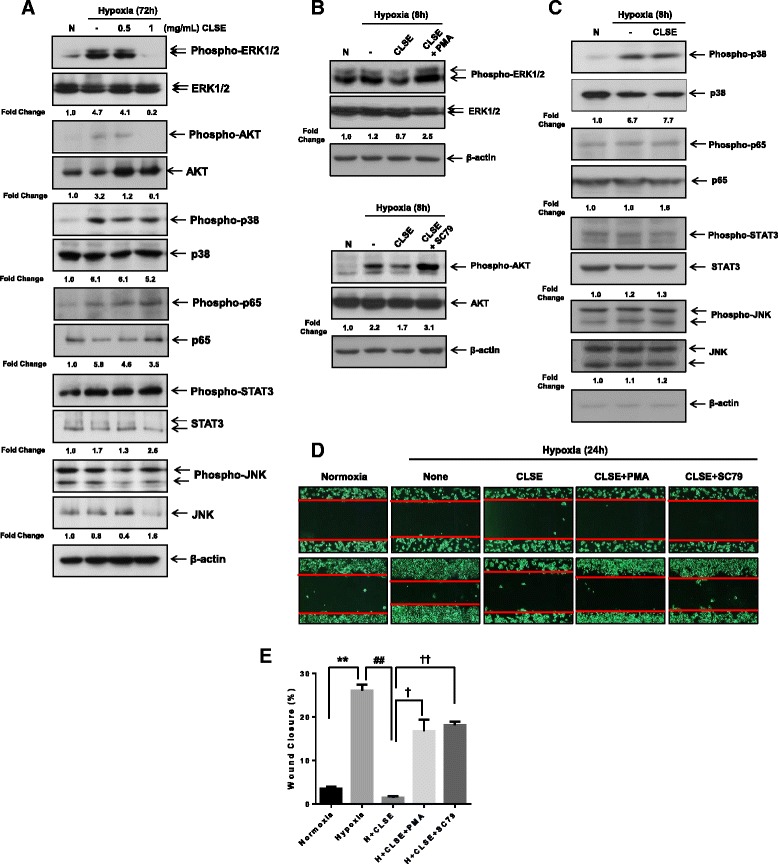
Effect of CLSE on signaling pathways under hypoxic conditions. **a**. CLSE-treated HCT116 cells under hypoxic conditions for 72 h were analyzed by western blot analysis. ERK1/2 and AKT phosphorylation were downregulated by CLSE treatment under hypoxic conditions. **b**, **c**. After cells were treated with CLSE (1 mg/mL), PMA (5 ng/mL) or SC79 (5 μg/mL) for 8 h under hypoxic conditions, signaling proteins were examined through western blot analysis. **d**. Representative scratch-wound images showing the effect of PMA and SC79 on the healing ability of HCT116 cells (magnification: 40×). After cells were treated with CLSE (1 mg/mL), PMA (10 ng/mL), or SC79 (2.5 μg/mL) for 24 h under hypoxic conditions, migrating cells were analyzed using ImageJ software. **e**. Percentage of HCT116 cells that migrated to the wound area following combined treatment with CLSE and PMA or SC79. ** *p* < 0.001 versus normoxia control group. ^##^ *p* < 0.01 versus hypoxia-only group. † *p* < 0.05, †† *p* < 0.01 versus hypoxia and CLSE treated group. Data are mean ± SD (*n* = 3). N, Normoxia; H, hypoxia; CLSE, *Coix lacryma-jobi var. ma-yuen* Stapf sprout extract; ERK, extracellular signal-regulated kinase; AKT, protein kinase B; PMA, phorbol 12-myristate 13-acetate; SD, standard deviation

To identify the cellular signaling pathways regulated by CLSE during colon cancer cell metastasis under hypoxic conditions, we examined the expression of signaling markers using Western blot analysis. Among the various signaling markers investigated, hypoxia-induced activation of ERK1/2 and AKT were downregulated by 1 mg/mL CLSE (Fig. 4a). To confirm whether the activation of ERK1/2 and AKT were repressed by CLSE in HCT116 cells, we performed western blot and scratch-wound healing assay at earlier stage in the presence of the ERK1/2 and AKT activators, PMA and SC79, respectively. As shown in Fig. 4b, inhibition of the ERK1/2 and AKT pathways by CLSE was reversed in CLSE-treated HCT116 cells given PMA or SC79. However, activation of other signaling proteins (p38, p65, STAT3, JNK) was not affected by CLSE treatment at an earlier stage (Fig. 4c). In addition, in the scratch-wound healing assay, PMA and SC79 could compromise the inhibitory effect of CLSE on the migration of HCT116 cells under hypoxic conditions by 57–71% and 64–77%, respectively (*p* < 0.05, *p* < 0.01) (Fig. 4d, e). Therefore, our results suggest that CLSE represses HCT116 cell migration through inactivation of the ERK1/2 and AKT pathways under hypoxic conditions.

### CLSE inhibits HUVEC tube formation under hypoxia

**Fig. 5:**
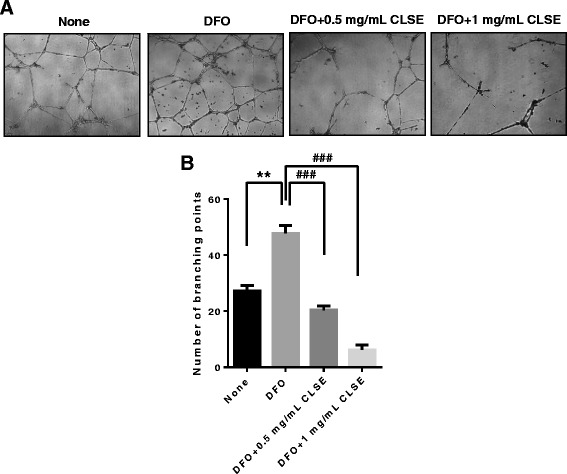
Effect of CLSE on HUVECs capillary tube formation. **a**. After HUVECs were seeded in matrigel-coated plates and incubated with conditioned media from HCT116 cells treated with DFO (100 μM) and CLSE (0.5, 1 mg/mL) for 24 h. Representative images showing HUVEC tube formation following treatment with conditioned media collected from CLSE-treated HCT116 cells (magnification: ×40). **b**. Number of branching points per unit area following treatment with conditioned media. ** *p* < 0.01 versus untreated control group. ^###^ *p* < 0.001 versus DFO-only group. Data are mean ± SD (*n* = 3). CLSE, *Coix lacryma-jobi var. ma-yuen* Strapt sprout extract; HUVECs, human umbilical vein endothelial cells; SD, standard deviation

The formation of new blood vessels (angiogenesis) is crucial for invasive tumor growth and metastasis. To demonstrate that CLSE affects angiogenesis, conditioned media, obtained from growing HCT116 cells in media supplemented with CLSE and/or DFO, was used to treat HUVEC cells. The media obtained from DFO-treated HCT116 cells augmented tube formation by HUVECs (*p* < 0.01), whereas conditioned media, obtained from CLSE and DFO-treated HCT116 cells, decreased tube formation by 55–91% (*p* < 0.001). These results suggest that CLSE may mediate its anti-angiogenic effect by regulating the secretion of angiogenic factors by colon cancer cells (Fig. 5).

## Discussion

The hallmarks of cancer are self-sufficiency of growth signals, insensitivity to anti-growth signals, limitless replicative potential, evading cell death (apoptosis), tissue invasion, metastasis, and sustained angiogenesis [2]. To investigate the anti-cancer effects of CLSE, we investigated the physiological effect of CLSE on cancer using human colon cancer cells.

In this study, we showed that CLSE had an anti-metastatic effect in colon cancer cells. Specifically, CLSE inhibited colon cancer cell migration, invasion, wound healing, and adhesion. In addition, conditioned media collected from CLSE-treated HCT116 cells had an inhibitory effect on HUVEC tube formation. On the other hand, in the case of the cervical cancer cell line HeLa, CLSE promoted apoptosis and arrested the cell cycle (in submission). Taken together, these results suggest that the mechanism on the anti-cancer effect of CLSE may be organ-specific.

Recently, many studies have tried to identify the active components of *Coix* and determine their mechanism of action. Specifically, neutral lipid isolated from endosperm of *Coix* inhibits the growth of pancreatic cancer cells [12], and the ethyl acetate fraction from ethanolic extraction of adlay testa has an inhibitory effect on the allergic response [13]. In addition, five compounds (coixspirolactam A, coixspirolactam B, coixspirolactam C, coixlactam, methyl dioxindole-3-acetate) isolated from *Coix* bran exhibit anti-proliferative effect on lung and colon cancer cells [9]. Because CLSE is obtained from a young sprout form *Coix*, we believe it to share a similar chemical composition with the mature plant. As such, it is highly likely that CLSE also contains coixspirolactams and methyl dioxindole-3-acetate, which may contribute to the anti-metastatic effects of CLSE.

Previous reports analyzed the proliferation of cancer cells or the expression of cell cycle regulatory proteins to demonstrate the anti-cancer effects of *Coix* under normal conditions. However, our study focused on the effects of the sprout extract of *Coix* on colon cancer cell metastasis and HUVEC tube formation under hypoxic conditions. Hypoxia, a characteristic feature of locally advanced solid tumors, has emerged as a pivotal factor for the tumor physiome because it can promote tumor progression and increase tumor resistance to therapy [14]. Migration, invasion, and adhesion of cancer cells result from the loss of epithelial markers and the degradation of basement membrane. Therefore, we expect that CLSE may have anti-metastatic effects via regulation of E-cadherin, vimentin, MMP-2, and MMP-9 in colon cancer cells, and plan to undertake these experiments in the future.

Phosphorylation-mediated activation of AKT and ERK signaling drives tumor invasion [15]. The ERK pathway controls cell migration, invasion, proliferation, and the induction of transcriptional programs [16]. Since AKT contributes to the development or progression of cancer, many consequences of hyperactive AKT signaling are considered hallmarks of cancer [17]. Our data showed that downregulation of ERK1/2 and AKT phosphorylation by CLSE was reversed in the presence of their activators, PMA and SC79 under hypoxic conditions (Fig. 4); therefore, it is likely that CLSE blocked the migration of colon cancer cells via inactivation of ERK1/2 and AKT under hypoxic conditions.

It has been reported in some animal research that *Coix* consumption might cause embryotoxicity and enhance uterine contractility during pregnancy [18]. Furthermore, some herbal medicines are reported to interfere with the efficacy and safety of conventional medicines [19]. Therefore, more research on the stability and efficacy of herbal supplements such as *Coix* is needed. In addition, our primary focus in this research was to explore the overall anti-cancer effects of CLSE, and we have succeeded in this endeavor. Further studies will not only reinforce this research, but will also contribute greatly to the field of oncology through detailed chemical profiling of CLSE and mechanistic studies of each component, as well as in vivo experiments using CLSE.

## Conclusions

CLSE can inhibit colon cancer cells migration, invasion, adhesion, and HUVEC tube formation in hypoxic conditions through inhibition of the ERK1/2 and AKT signaling pathways. However, further study is necessary to reveal the possible mechanism of the anti-metastatic effect of CLSE and its active compounds under hypoxic conditions.

## Abbreviations

AKT: Protein kinase B
CCK-8: Cell Counting Kit-8
CLSE: *Coix lacryma-jobi var. ma-yuen* Stapf sprout extract
DFO: Deferoxamine
ERK: Extracellular signal-regulated kinase
FBS: Fetal bovine serum
HUVECs: Human umbilical vein endothelial cells
JNK: c-Jun N-terminal kinase
KLT: Kanglaite®
MTT: 3-(4,5-Dimethylthiazol-2-yl)-2,5-diphenyltetrazolium bromide
PBS: Phosphate buffered saline
PMA: Phorbol 12-myristate 13-acetate
SD: Standard deviation
SDS: Sodium dodecyl sulfate
STAT3: Signal transducer and activator of transcription 3
WST-8: 2-(2-methoxy-4-nitrophenyl0–3-(4-nitrophenyl)-5-(2,4-disulfophenyl)-2H–tetrazolium, monosodium salt
